# The prognostic value of HPV combined p16 status in patients with anal squamous cell carcinoma: a meta-analysis

**DOI:** 10.18632/oncotarget.23545

**Published:** 2017-12-21

**Authors:** Guorui Sun, Xiaoyuan Dong, Xiaolong Tang, Hui Qu, Hao Zhang, Ensheng Zhao

**Affiliations:** ^1^ Department of Gastrointestinal Surgery, Qilu Hospital of Shandong University, Jinan, Shandong, P.R. China; ^2^ Department of Hematology, Qilu Hospital of Shandong University, Jinan, Shandong, P.R. China

**Keywords:** HPV, p16, anus carcinoma, prognosis, meta-analysis

## Abstract

Human papillomavirus (HPV) DNA and p16 expression have been identified to be related to the progression of anal squamous cell carcinoma (ASCC). However, the prognostic relevance of combined detection, particularly HPV-/p16+ and HPV+/p16- signatures, is unknown. A meta-analysis of epidemiologic studies was therefore conducted to address this issue. Data were collected from studies comparing overall survival (OS) and disease-free survival (DFS) / disease-specific survival (DSS) / relapse-free survival (RFS) / progression-free survival (PFS) in ASCC patients with HPV and p16 status. The electronic databases of MEDLINE and EMBASE were searched from their inception till 31 May 2017. Study-specific risk estimates were pooled using a fixed-effects model for OS and DFS/DSS/RFS/PFS. Four studies involving a total of 398 ASCC cases were included in this meta-analysis. The pooled results showed that HPV+/p16+ cancers were significantly associated with improved OS (HR = 0.30, 95% CI: 0.17–0.51) and DFS/DSS/RFS/PFS (HR = 0.23, 95% CI: 0.14–0.36). However, patients with HPV-/p16+ or HPV+/p16- do not have a comparably good prognosis compared with HPV+/p16+ patients. The meta-analysis indicated that concomitant detection of HPV-DNA and p16 expression may be of prognostic or therapeutic utility in the evaluation of factors contributing to ASCC. Testing tumor specimens for HPV-DNA and p16 expression might indirectly affect treatment decisions.

## INTRODUCTION

The incidence rate of Anal squamous cell carcinoma (ASCC) ranges from 1.0 to 2.5 per 100,000 population in a lot of developed countries, which is an rare malignancy of the anal canal and perianal skin area [1]. The annual incidence increases by about 2%, especially in females [2–4].

Even though many risk factors for the development of ASCC have been identified, ASCC is known to be strongly linked with a small double-stranded DNA virus, the human papillomavirus (HPV), known for its role in the development of head and neck squamous cell carcinoma (HNSCC), cervical cancer and other gynaecological cancers [5–7]. The prevalence in ASCC for high-risk HPV (HR-HPV) types, which are associated with carcinogenesis, ranges from 70 percent to 100 percent, depending on the sensitivity of the method used for HPV detection and the studied population [8–10].

One study reported that HPV-positive cervical cancer patients receiving radiotherapy have significantly better survival [11]. Other studies for HNSCC also shown that HPV-positive patients had a better prognosis than those HPV-negative [12–15]. Anus can be infected with these viruses the same way as the oral cavity, pharynx, and tonsils do; it is assumed that histological similarities of squamous epithelia between anus and the head and neck suggest clinical features.

The value for prognostic role of the HPV status has already been studied in ASCC patients. However, the sole detection of HPV DNA may misclassify cancers as being associated with HPV, since it does not prove the overexpression of viral oncogenes and thus the transformation induced by HPV [16]. As a result, additional biomarkers are being developed to refine the identification of HPV-associated tumors to achieve clinically acceptable accuracy. On this point, the cyclin-dependent kinase inhibitor 2A (CDKN2A), better known as p16^INK4a^ (p16), appeared to be the best validated candidate because of its association with high-risk HPV infection. This cyclin-dependent kinase inhibitor is normally repressed by a phospho-retinoblastoma protein (pRB)/transcription factor E2F complex, but this suppression is inhibited by the high-risk E7 oncoprotein of HPV, resulting in an overexpression of p16^INK4a^ [17, 18]. According to this, several studies have indicated that p16^INK4a^ detection by immunohistochemistry (IHC) is predictive for a significantly improved response to treatment with RT/CRT and a more favourable prognosis in patients with HPV-associated malignancies [19–21].

Therefore, we conducted the meta-analysis to assess the combined effects of HPV status and p16 expression on overall survival (OS) and disease-free survival (DFS)/ disease-specific survival (DSS/relapse-free survival (RFS)/progression-free survival (PFS) in patients of ASCC.

## RESULTS

### Study selection

**Figure 1:**
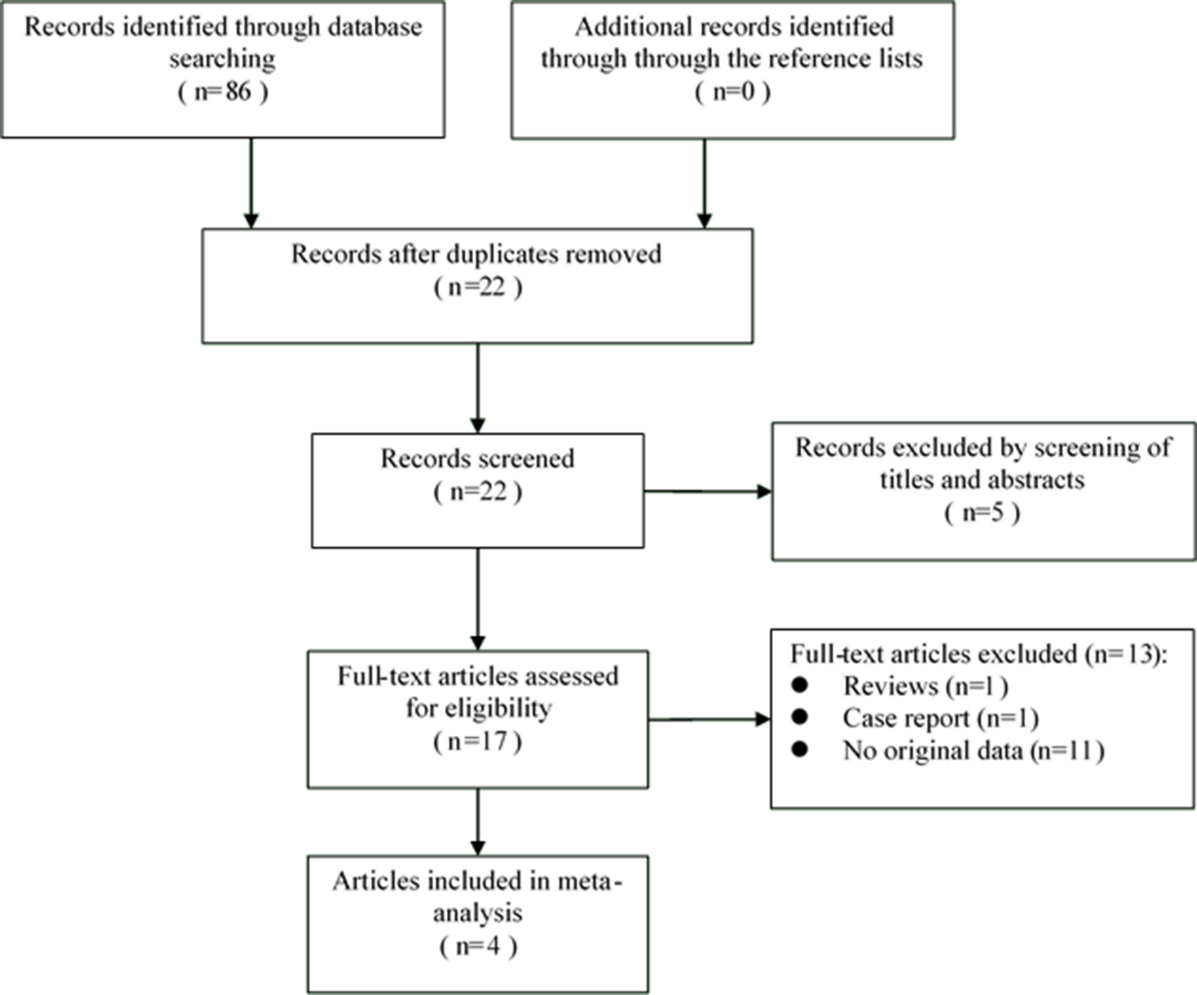
The PRISMA flow diagram of systematic literature search

86 citations were generated from the search strategy. Through the literature search and browsing titles and/or abstracts, 17 papers were identified as potentially relevant (Figure 1). 13 of these 17 articles were subsequently excluded from our study due to several reasons, including 1 was review, 1 was case report, and 11 that did not provide *HR*s or *CI*s. A total of 4 prospective studies was included in the final meta-analysis [16, 22–24].

### Characteristics of the selected studies

Table 1 summarized the each characteristics of the included 4 studies. The total number of patients included in themeta-analysis was 398, involingsin OS and DFS/DSS/RFS/PFS in both 4 studies. *HR*s and 95% *CI*s were directly extracted from the survival curve of two studies [16, 23]. One study [22] did not providespecific data for follow-up. The median follow-up period for all included studies ranged from 40 to 54 months. The prevalence of HPV varied from 67.9% to 95.8%, and the prevalence of p16 varied from 65.3% to 90.7%.

**Table 1:** Characteristics of the included studies

First author	Year	Period of recruitment	Country	Study design	Stage	No. of patients	Genotype (s)	HPV + ve N (%)	p16 + ve N (%)	Age, y	Treatment	DNA/p16 method	Median follow-up period (months)	Survival analysis	Hazard ratio
Koerber SA	2014	2000–2011	Germany	Retrospective	I-III	90	24 types^*^	75 (83.3)	75 (83.3)	55 (22–94)	CRT or R	PCR/IHC	48.6 (2.8–169.1)	OS/PFS	SC
Rödel F	2014	NA	Germany	Prospective	I-IV	95	28 types^#^	91 (95.8)	62 (65.3)	NA	CRT	PCR/IHC	40 (1–264)	OS/CSS	SC
Meulendijks D	2015	2003–2011	Netherlands	Prospective	I-III	107	28 types^#^	93 (86.9)	97 (90.7)	60 (34–86)	CRT or R	PCR/IHC	NA	OS/DFS	Adjusted
Mai S	2015	1990–2012	Germany	Prospective	I-III	106	24 types^*^	72 (67.9)	74 (69.8)	59.5(31–86)	CRT	PCR/IHC	54 (5–205)	OS/DFS	Unadjusted

Abbreviations: HPV + ve, human papillomavirus positive; p16 + ve, p16 positive; R, radiotherapy; CRT, chemo-radiotherapy; PCR, polymerase chain reaction; IHC, immunohistochemistry; OS, overall survival; DFS, disease-free survival; CSS, cancer-specific survival; RFS, relapse-free survival; PFS, progression-free survival; SC, survival curve; NA, not available.

^*^ Including 15 high-risk types (HPV 16, 18, 31, 33, 35, 39, 45, 51, 52, 56, 58, 59, 68, 73, 82), 3putative high-risk types (HPV 26,53,66), and 6 low-risk types(HPV 6, 11, 42, 43, 44, 70); ^#^ Including 20 high-risk types (HPV 16, 18, 26, 31, 33, 35, 39,45, 51, 52, 53, 56, 58, 59, 66, 68, 69, 70, 73, 82) and 8 low-risk types (HPV 6, 11,40, 43, 44, 54, 71, 74).

### Quality assessment

The mean quality score of individual studies was 71.02% (range from 56.82% to 79.55%), indicating that the studies included in this meta-analysis were with relatively high quality (Table 2).

**Table 2:** Methodological assessments of the studies included in the meta-analysis

First author	Global score (%)	Scientific desigh (/10)	Laboratory methodology (/14)	Generalizability (/12)	Results analysis (/8)
Koerber SA	75.00	8	10	10	5
Rödel F	72.23	9	10	8	5
Meulendijks D	79.55	7	12	10	6
Mai S	56.82	6	6	8	5

### Results of the meta-analysis

The heterogeneity test, in particular, had a very low degree of heterogeneity among included studies, so that the pooled HR was obtained by a fixed effects model.

### Overall survival

**Figure 2:**
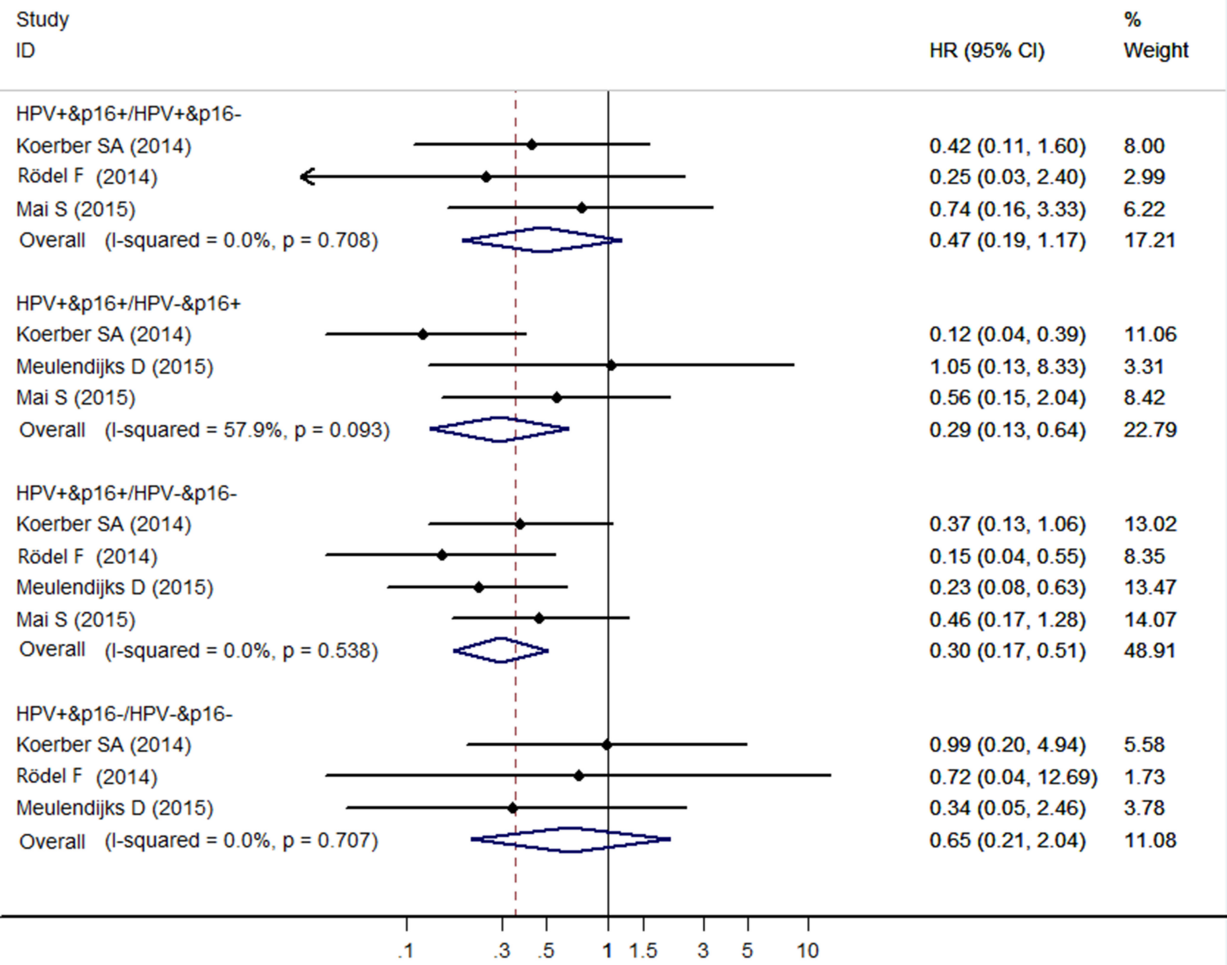
Forest plot for the association between HPV/p16 status and OS in ASCC patients

The *HR* pooled from the 3 individual effect estimates comparing HPV+/p16+ to HPV+/p16- cancers was 0.47 (95% *CI*: 0.19–1.17), which was not significantly correlated with OS. The meta-analysis showed a significant association for OS comparing HPV+/p16+ to HPV-/p16+ cancers the *HR* (95% *CI*) being 0.29 (95% *CI*: 0.13–0.64) from the 3 individual effect estimates. Compared with HPV-/p16- cancers, patients with HPV +/p16+ cancers had significant improved OS (*HR* = 0.30, 95% *CI*: 0.17–0.51), and patients with HPV+/p16- cancers had no significant improved OS (*HR* = 0.65, 95% CI: 0.21–2.04) (Figure 2).

### DFS/DSS/RFS/PFS

**Figure 3:**
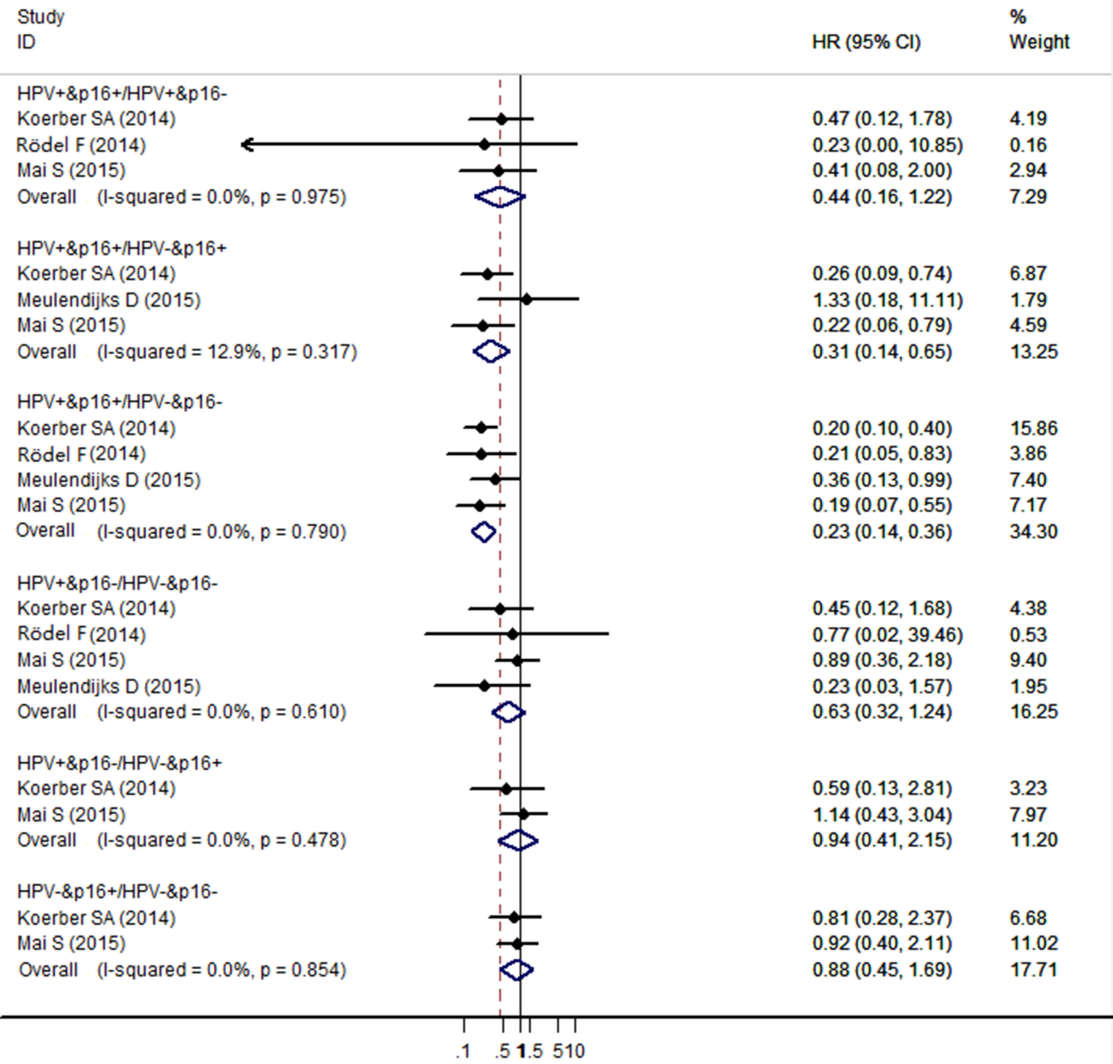
Forest plot for the association between HPV/p16 status and DFS/DSS/RFS/PFS in ASCC patients

The meta-analysis showed no significant association for DFS/DSS/RFS/PFS comparing HPV+/p16+ to HPV-/p16+ cancers the *HR* (95% *CI*) being 0.44 (95% *CI*: 0.16–1.22) from the 3 individual effect estimates. Compared with HPV-/p16+ cancers, patients with HPV+/p16+ cancers had significant improved DFS/DSS/RFS/PFS (*HR* = 0.31, 95% *CI*: 0.14–0.65), and patients with HPV+/p16- cancers had no significant improved DFS/DSS/RFS/PFS (*HR* = 0.94, 95% *CI*: 0.41–2.15). Compared with HPV-/p16- cancers, patients with HPV+/p16+ cancers had significant improved DFS/DSS/RFS/PFS (*HR* = 0.23, 95% *CI*: 0.14–0.36), patients with HPV+/p16- cancers had no significant improved DFS/DSS/RFS/PFS (*HR*=0.63, 95% *CI*: 0.32–1.24), and patients with HPV-/p16+ cancers had no significant improved DFS/DSS/RFS/PFS (*HR* = 0.88, 95% *CI*: 0.45–1.69) (Figure 3).

## DISCUSSION

This is the first systematic review of the prognostic impact of HPV status together with p16 expression in ASCCs. In this study, we find that HPV and p16 status are strong predictors for OS and DFS/DSS/RFS/PFS. Pooled effect estimates among included studies demonstrated that ASCC patients with HPV-/p16- or HPV-/p16+ was considerably inferior compared with those patients with HPV+/p16+, suggesting that, to evaluating factors contributing to ASCC, HPV and p16 status especially HPV status, may be of therapeutic or prognostic utility.

In the late 1980s, it's the first time to reported the relationship between HPV infection and the incidence of ASCC [25–27]. After then, the association between HPV infection and ASCC prognosis have been reported by a cumulative number of studies. However, the resulting studies were inconsistent. Since overexpression of p16 is not entirely limited to HPV-related transformation, it is important to analyze the combined status of HPV-DNA/p16 in order to more accurately identify cancers associated with HPV. S prognosis [28, 29]. However, data is limited in the assessment of the coefficient of exposure to HPV-DNA and p16 expression on the survival in patients of ASCC, which is of interest to us. CC. We showed that HPV+/p16+ ASCCs had a 70% reduction in OS and a 77% reduction in DFS/DSS/RFS/PFS, in comparison to HPV-/p16- ASCCs. HPV+/p16+ patients seemed to be sufficiently treated with current radiation therapy doses concomitantly with standard chemotherapy.

It is important to note that HPV-/p16+ patients do not have a comparably good prognosis as HPV+/p16+ patients, which is probably because the HPV-/p16+ tumors are not induced by HPV. Cancers associated HPV often have a viral sequence integrated into cancer cells genome. E6 and E7 are two early structural genes of the HPV. Through inactivation of p53 and the retinoblastoma protein (pRb), a negative regulator of the cyclin-dependent kinase inhibitor p16 and thus leads to upregulation of p16 [12, 30], the E6 and E7 proteins contribute to the genetic instability. Interfering changes in *TP53* were reported in HPV-/p16- tumours (80%) and HPV-/p16+ tumours (33%), compared only sporadically to HPV+/p16+ tumours (6%) [22]. It is not surprising that *TP53* mutations are only sporadically found in HPV+ tumours, as the HPV oncoprotein E6 inhibits p53 function by targeting it for ubiquitination and degradation. An additional mutation in *TP53* would, therefore, not be necessary for these tumours to evolve. The apparent lower frequency of *TP53* mutations in HPV-/p16+ tumours could be explained by aberrations in other tumour suppressor proteins, which could be investigated in future studies. The loss of p53 function was related to resistance to radiotherapy [31–33]. It is therefore conceivable that patients with HPV- tumours have a lower treatment response level due to a higher frequency of disrupted p53 function (via *TP53* mutations). Other tests, such as HPV E6/E7 mRNA tests, can be evaluated in future studies in comparison with combined HPV DNA/p16 detection. However, HPV DNA and p16 tests are easy to perform and widely used, and are therefore accepted candidate prognostic markers.

The strengths including: a) the current analysis is the first to study the prognostic impact of HPV status together with p16 expression in ASCCs, and b) we used a strict inclusion and exclusion criterions, fully outcomes of interest (OS and DFS/DSS/RFS/PFS) and an advanced meta-analysis of *HR* for survival. The limitations including: a) a comparison of all combined HPV-DNA- and p16- subgroups in this meta-analysis is limited due to the few studies included with HPV-/p16+ and HPV+/p16- cancers. b) only English studies were included in the meta-analysis, which might resulting in language bias, and c) the adjusted *HR*s was only reported in one study, which might introduce residual confounding caused by other prognostic factors.

## MATERIALS AND METHODS

### Literature search strategy

A systematic search up to 31 May 2017 was conducted in MEDLINE (via PubMed) and Excerpta Medica database (EMBASE) to identify relevant articles. Search terms included “human papillomavirus OR HPV”, “p16 OR CDKN2 OR INK4A”, “anal cancer OR anal neoplasms OR anal carcinoma” combined with “prognosis OR prognostic OR survival”. Additional relevant references cited in retrieved articles were also evaluated.

### Inclusion and exclusion criteria

All papers were reviewed by two authors (X.T. and H.Q.) independently. Uncertainties and discrepancies were resolved by consensus after discussing with a senior researcher (G.S.). All studies included in the final meta-analysis satisfied the following criteria: (a) patients were pathologically diagnosed as ASCC; (b) OS or DFS/DSS/RFS/PFS as the outcome of interest; (c) reported *HR*estimates with their corresponding 95% *CI* (or sufficient data to calculate of these effect measure), and (d) English articles. If the study was reported in duplication, the one published earlier or provided more detailed information was included. Review articles and editorials were included if they contained original data. Abstracts were excluded.

### Quality assessment

The quality of each study was evaluated in accordance with the revised ELCWP scoring scale described by Steel [34]. Each item was assessed using an ordinal scale (possible values: 2, 1, 0). The overall score evaluated several dimensions of the methodology, grouped into four main categories: (1) scientific design: 0–10; (2) laboratory methodology: 0–14; (3) generalizability: 0–12; (4) results analysis: 0–8. The total scores ranged from 0 to 44. The final scores were expressed as percentages, ranging from 0% to 100%, higher values indicated a better methodological quality.

### Data extraction

Two of the authors (X.T. and H.Q.) performed the data extraction from each article and discrepancies were resolved by consensus. For studies meeting our inclusion criteria, a standardized data extraction form was used to extract the following data: the first author's name, year of publication, country of origin, study design, period of enrollment, the length of follow-up, characteristics of the studied population (sample size, age, stage of disease and treatment method), HPV detection methods, p16 detection methods, and *HR* estimates for OS or DFS/DSS/RFS/PFS with corresponding 95% *CI*s. When data for *HR* was not available, we extracted the total numbers of observed deaths and the numbers of patients in each group to calculate *HR*. [35]. Data were extracted by Engauge Digitizer version 4.1 (http://digitizer.sourceforge.net/) from the graphical survival plots when data were only available as Kaplan-Meier curves, [36]. then the estimation of the *HR* was performed by the described method [35].

### Statistical analysis

The *HR* with 95% *CI* was used to compute the pooled HPV status combined p16 expression and the OS or DFS/DSS/RFS/PFS in ASCC patients. A fix-effect or random-effect model was used to pool the data, based on the Mantel–Haenszel method [37]. and the DerSimonian and Laird method, [38]. respectively. These two models provide similar results when between-studies heterogeneity is absent; otherwise, random-effect model is more appropriate.

Cochrane *Q* test *(P* < 0.10 indicated a high level of statistical heterogeneity) and *I*^2^ (values of 25%, 50% and 75% corresponding to low, moderate and high degrees of heterogeneity, respectively) was used to assess the heterogeneity between eligible studies, which test total variation across studies that was attributable to heterogeneity rather than to chance [39].

All statistical analyses were performed using STATA version 12 for Windows (StataCorp LP, College Station, TX, USA). A two-tailed *P* < 00.05 was considered statistically significant.

## CONCLUSIONS

In conclusion, concomitant detection of HPV-DNA and p16 expression represents a prognostic marker in ASCC patients. Escalating treatment options for HPV-/p16- cancers and de-escalating therapy for ASCCs with HPV+/p16+ could be considered with the order to generate better outcome and fewer side effects related to treatment. An overexpression of p16 without HPV infection seem to have an unfavorable prognosis and may therefore require intensification of treatment for ASCCs. In this rule, prospective trials are mandatory to further determine the predictive joint-role of HPV-DNA and p16 expression in ASCC patients.
